# Oxidized LDL Triggers Pro-Oncogenic Signaling in Human Breast Mammary Epithelial Cells Partly via Stimulation of MiR-21

**DOI:** 10.1371/journal.pone.0046973

**Published:** 2012-10-16

**Authors:** Magomed Khaidakov, Jawahar L. Mehta

**Affiliations:** Central Arkansas Veteran Healthcare System and the University of Arkansas for Medical Sciences, Little Rock, Arkansas, United States of America; Tor Vergata University of Rome, Italy

## Abstract

Dyslipidemia and obesity are primary risk factors for the development of atherosclerosis and are also epidemiologically linked to increased susceptibility to a variety of cancers including breast cancer. One of the prominent features of dyslipidemia is enhanced production of oxidized LDL (ox-LDL), which has been shown to be implicated in key steps of atherogenesis including inflammatory signaling and proliferation of vascular cells. In this study we analyzed the effects of ox-LDL in human mammary epithelial cells (MCF10A). MCF10A cells avidly internalized dil-ox-LDL and exhibited increased proliferative response to ox-LDL within the range of 1–50 µg/ml in a dose-dependent manner. Treatment of cells with 20 µg/ml ox-LDL for 2 and 12 hours was associated with upregulation of LOX-1 and CD36 scavenger receptors while MSR1 and CXLC16 receptors did not change. Ox-LDL-treated cells displayed significant upregulation of NADPH oxidases (subunits P22^phox^ and P47^phox^), lipoxygenases-12 and -15, and cytoplasmic, but not mitochondrial, SOD. Ox-LDL also triggered phosphorylation of IκBα coupled with nuclear translocation of NF-κB and stimulated p44/42 MAPK, PI3K and Akt while intracellular PTEN (PI3K/Akt pathway inhibitor and target of miR-21) declined. Quantitative PCR revealed increased expression of hsa-miR-21 in ox-LDL treated cells coupled with inhibition of miR-21 target genes. Further, transfection of MCF10A cells with miR-21 inhibitor prevented ox-LDL mediated stimulation of PI3K and Akt. We conclude that, similarly to vascular cells, mammary epithelial cells respond to ox-LDL by upregulation of proliferative and pro-inflammatory signaling. We also report for the first time that part of ox-LDL triggered reactions in MCF10A cells is mediated by oncogenic hsa-miR-21 through inhibition of its target gene PTEN and consequent activation of PI3K/Akt pathway.

## Introduction

Association between obesity and breast cancer is well established and is likely based on multiple mechanisms [1], [2]. Experimental evidence suggests involvement of paracrine interactions between epithelial cells and adipocytes, metabolic syndrome-mediated alterations in insulin signaling and conversion of androgens into estrogens in obese postmenopausal women [3]–[5]. There are, however, other possible mechanisms that may be implicated in dyslipidemia-mediated susceptibility of mammary epithelium to oncogenic transformation.

In particular, oxidatively modified low density lipoprotein (ox-LDL) has been identified as one of the primary factors responsible for atherogenesis [6]–[8]. LDL oxidation results in formation of various biologically active molecules (many of them with mutagenic properties) and brings about fundamental change with regard to ox-LDL particle payload and destination. Rather than being eliminated via reverse cholesterol transport machinery using LDL receptor, ox-LDL is recognized and captured by scavenger receptors abundant in peripheral tissues.

In vascular cells, internalization of ox-LDL has been shown to trigger signaling events resulting in overproduction of reactive oxygen species, inflammation and proliferation [9], [10]. Various epithelial cells have also been shown to express ox-LDL specific scavenger receptors [11], [12]. Recent comparison of transformation transcriptomes of normal mammary epithelial cells MCF10A and primary fibroblasts highlighted the role of a major ox-LDL scavenger receptor LOX-1 in transformation through its involvement in NF-κB-dependent pro-inflammatory and hypoxic signaling [13]. In a previous report [14], we showed that overexpression of LOX-1 in normal epithelial and breast cancer cells results in upregulation of NF-kB and its target oncogenes accompanied with increased proliferation and migration of these cells. The purpose of the present study was to further elucidate mechanisms underlying effects of ox-LDL on mammary epithelial cells.

## Materials and Methods

### Cells and reagents

Normal human mammary epithelial cells MCF10A cells and corresponding MEGM growth medium with complement SingleQuots were purchased from the American Tissue Culture Collection (ATCC, Manassas, VA). High T-bar oxidized-LDL (64.2 nmoles MDA/mg protein) and Dil-ox-LDL were purchased from Biomedical Technologies (Stoughton, MA). Source of antibodies to LOX-1, CD36, CXLC16, MSR1, lipoxygenase-12 and lipoxygenase-15 was AbCam (Cambridge, MA), antibodies to SOD1 and SOD2 was Enzo Life Sciences (Exeter, UK), antibodies to PI3K, PTEN, and Akt was Cell Signaling Technology (Danvers, MA), and antibodies to NADPH oxidase (subtypes P47^phox^ and P22^phox^) was Santa Cruz (Santa Cruz, CA).

### Quantitative PCR

Total RNA was purified using the RNeasy mini kit (Qiagen, Valencia, CA), and cDNA was synthesized using the iScript cDNA synthesis kit (Bio-Rad) with 0.5 µg of total RNA according to the manufacturer's recommendations. qPCR was performed with pre-designed primers selected from PrimerBank [15] and ordered from Integrated DNA Technologies (Coralville, IA). RT qPCR was performed using the Applied Biosystems 7900 real-time PCR system. All qPCR reactions were carried out in a final volume of 15 µl containing 1X of SYBR Green PCR Master Mix (Applied Biosystems, Carlsbad, CA), 300 nM of each gene specific primers, 100 ng cDNA, in sterile deionized water. The standard cycling condition was 50°C for 2 min, 90°C for 10 min, followed by 40 cycles of 95°C for 15 s and 62°C for 1 min. The results were analyzed using SDS 2.3 relative quantification manager software. The comparative threshold cycles values were normalized for GAPDH reference genes. qPCR was performed in triplicate to ensure quantitative accuracy.

### MicroRNA qPCR

Primers for RT-PCR reaction and qPCR analysis were purchased from Applied Biosystems (Foster City, CA), and analysis was performed in triplicates for each data point according to manufacturer's instructions.

### Viability assessment

The evaluation of cytotoxicity was performed using cell count (trypan blue exclusion assay) and MTT assay (ATCC) based on reduction of yellow MTT (3-(4,5-Dimethylthiazol-2-yl)-2,5-diphenyltetrazolium bromide) to purple formazan in the metabolically active mitochondria of living cells. HCAECs were seeded into 96-well plates and allowed to reach 100% confluence. Confluent cultures were exposed to various concentrations of ox-LDL for 12 hours. Upon completion of exposure, growth medium was replaced and MTT (final concentration 5 mg/ml) added. After full development of color, formazan was solubilized and absorbance was measured at 570 nm.

### Dil-ox-LDL uptake

Cells were incubated with 1 µg/ml Dil-ox-LDL for 2 hours. Upon completion of incubation, cells were gently washed with 1× PBS three times to remove free Dil-ox-LDL and analyzed using fluorescent microscope.

### Other methods

Western blotting and immunostaining were performed using standard protocols. Hsa-miR-21 inhibitor was purchased from Qiagen (Valencia, CA) and introduced into the cells (50 mM final concentration) using HiPerfect transfection reagent (Qiagen) according to manufacturer's instructions.

### Statistical analysis

Data are presented as means ± standard deviation (SD). The statistical analysis was performed with SPSS 11.5 software. Multiple comparisons were analyzed by one-way ANOVA. A p value<0.05 was considered to be significant.

## Results

### Ox-LDL stimulates cell proliferation and expression of scavenger receptors

**Figure 1:**
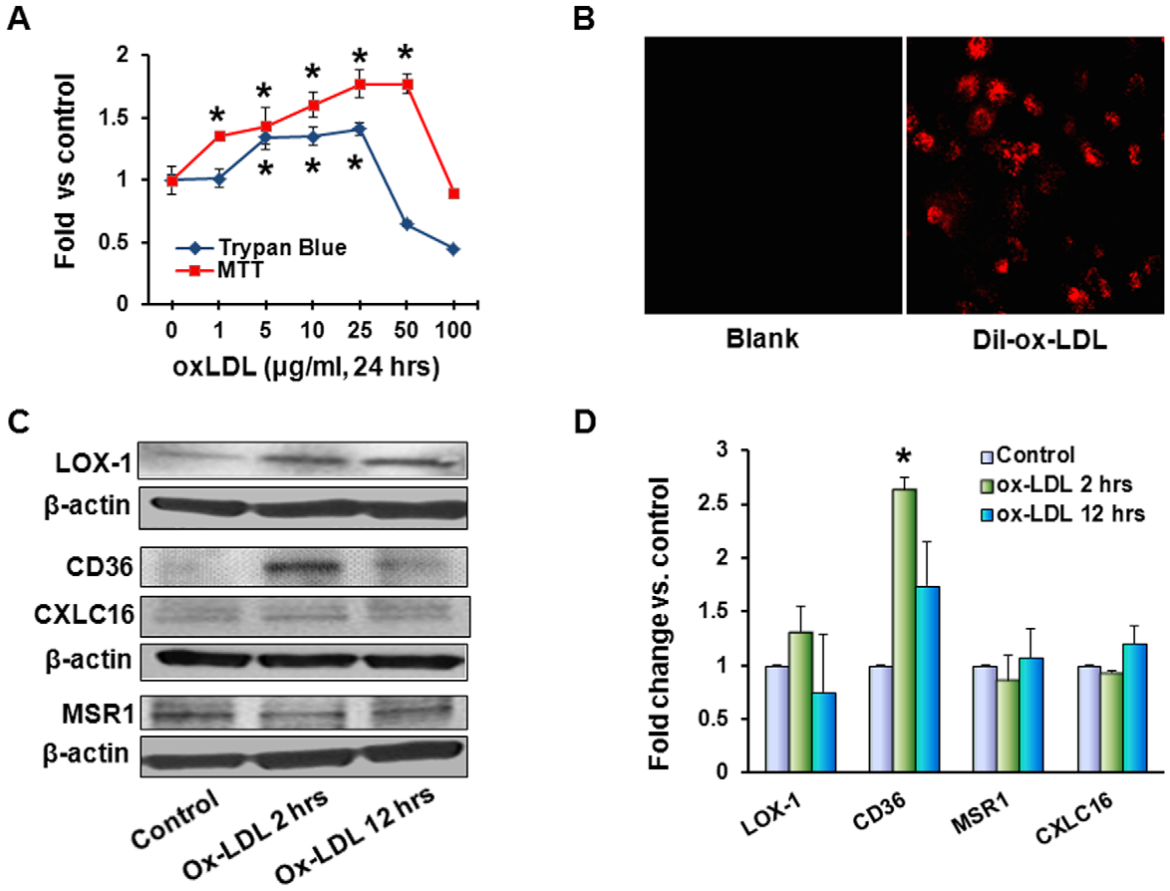
Ox-LDL stimulates cell proliferation and expression of scavenger receptors. (**A**) – For evaluation of proliferative response to ox-LDL measured by cell count and MTT assay, MCF10A cells were exposed to different concentrations of ox-LDL (1–100 µg/ml) for 24 hours; (**B**) – uptake of Dyl-ox-LDL by MCF10 A cells was visualized by fluorescent microscopy after 2 hours of incubation with 1 µg/ml Dil-ox-LDL; (**C**) – Western blot for scavenger receptors and (**D**) – graph depicting relative densities of bands normalized for β-actin in relation to control (fold change). (*) – significant difference (p<0.05) compared to control. For these experiments, MCF10A cells were treated with 20 µg/ml ox-LDL for 2 and 12 hours.

Treatment of cells with ox-LDL (1 to 25 µg/ml) for 24 hours had a dose-dependent stimulatory effect on cell proliferation evaluated by the cell count and MTT assay. In cultures exposed to ox-LDL, cell number exhibited robust stimulation of proliferation within 1–5 µg/ml, still positive but shallower response within 10–25 µg/ml range and decline in cell number at 50 µg/ml. In MTT assay, mitochondrial activity continued to rise at 1–25 µg/ml concentrations, reached plateau at 50 µg/ml and declined at 100 µg/ml (Figure 1A). Mammary epithelial cells MCF10A vigorously internalized Dil-ox-LDL (Figure 1B). Western blot analysis for main scavenger receptors involved in ox-LDL uptake revealed increase in LOX-1 expression (1.3-fold, p = 0.06) detectable after 2 and 12 hours of exposure to 20 µg/ml ox-LDL (Figures 1C and D). CD36 increased more than 2-fold after 2 hours of incubation (p<0.03) followed by a decline, whereas macrophage scavenger receptor 1 (MSR1) as well as chemokine (C-X-C motif) ligand 16 (CXCL 16) remained unaffected.

### Ox-LDL exposure is followed by upregulation of NADPH oxidases, LDL modifying enzymes and SOD1

**Figure 2:**
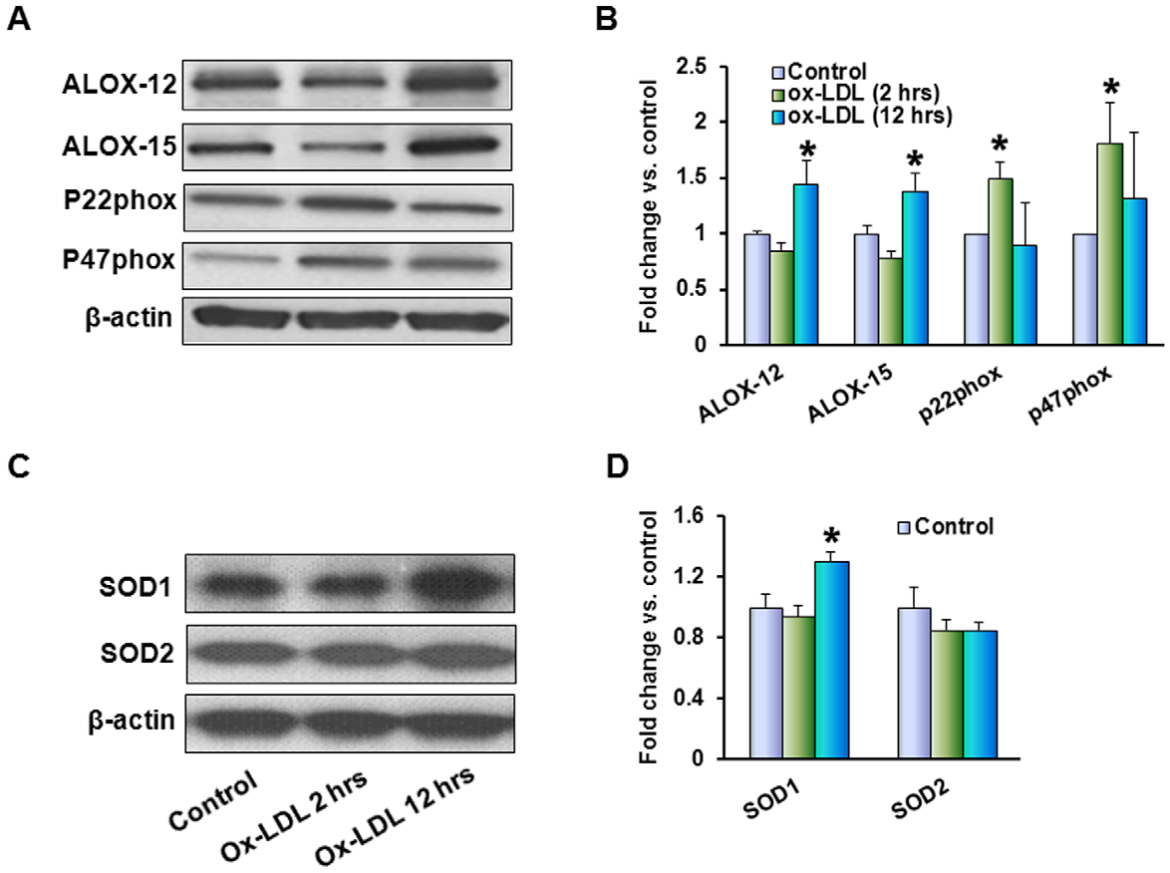
Ox-LDL exposure is followed by upregulation of NADPH oxidase, LDL modifying enzymes and SOD1. (**A**) – Western blots for Lipoxygenases-12, lipoxygenase -15-2 and P22^phox^ and P47^phox^ subunits of NADPH oxidase (treatment with 20 µg/ml ox-LDL for 2 and 12 hours); (**B**) – corresponding graphs depicting relative densities of bands normalized for β-actin in relation to control (fold change); (**C**) – Western blots for SOD1 and SOD2 (similar conditions) and (**D**) – corresponding graphs depicting relative densities of bands normalized for β-actin in relation to control (fold change). (*) – Significant difference (p<0.05) compared to control.

In order to verify that in ox-LDL modifies epithelial cells in a manner similar to that reported for vascular endothelial cells, we examined the expression of NADPH oxidase subunits P22^phox^ and P47^phox^. Content of both proteins increased after 2 hours of exposure of MCF10A cells to ox-LDL, bur the increase was transient (Figures 2A and B). In contrast, lipoxygenases-12 and -15 – identified as main contributors to LDL oxidation by vascular cells [16] – showed a substantial increase only at 12 hours. In a manner similar to lipoxygenases, SOD1 content did not change at 2 hrs but increased significantly at 12 hours after exposure to ox-LDL whereas SOD2 remained unaffected (Figures 2C and D).

### Ox-LDL triggers NF-kB and MAPK signaling

**Figure 3:**
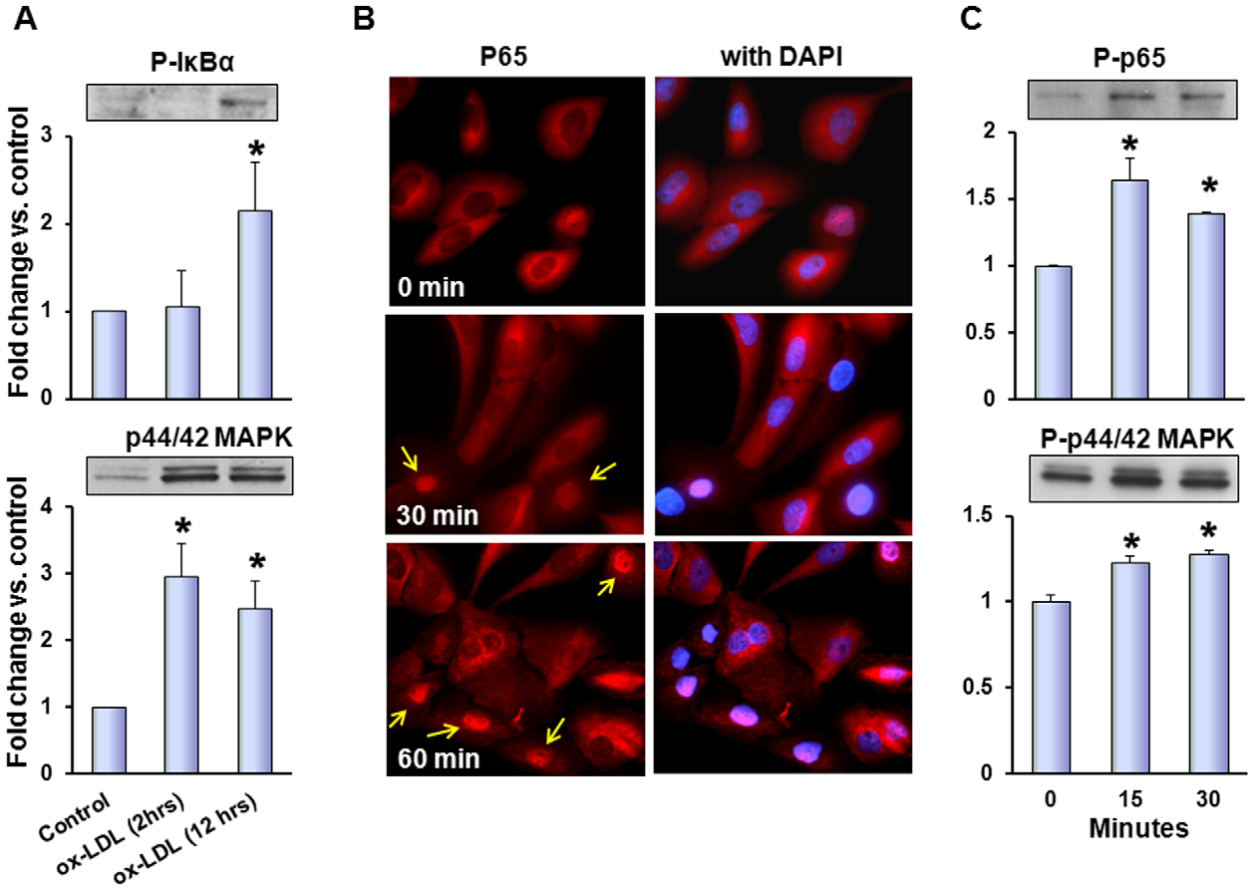
Ox-LDL triggers NF-kB and MAPK signaling. (**A**) – Western blots for phospho-IκBα and p44/42 MAPK with corresponding graphs depicting relative densities of bands normalized for β-actin in relation to control (fold change); (**B**) – Immunostaining for phosphor-p65 (NFκB) in MCF10A. Note that cells exposed to ox-LDL display translocation of protein to the nucleus (arrows) within 30 minutes of exposure and that percentage of cells with translocated p65 increases with time. (**C**) - Western blots for phospho-p65 and phosphor-p44/42 MAPK within short (30 min) exposure to 20 µg/ml ox-LDL with corresponding graphs depicting relative densities of bands normalized for β-actin in relation to control (fold change); (*) – Significant difference (p<0.05) compared to control.

Treatment with ox-LDL was accompanied with sharp and lasting stimulation of MAPK p44/42 - a marker of cell proliferation - at 2 hours of incubation (Figure 3B). At later time point, we also detected increase in phosphorylation of NF-κB inhibitory subunit IκBα (Figure 3A). Within shorter time frame, phosphorylation of p65 and p44/42 MAPK became obvious as early as 15 minutes following exposure to 20 µg/ml ox-LDL (Figure 2C). Activation of NF-κB pathway was confirmed by translocation of p65 protein to the nucleus which became visible within 30 minutes (Figure 2B, arrows).

### Ox-LDL increases expression of miR-21 accompanied with PI3K/Akt upregulation and inhibition of target genes

**Figure 4:**
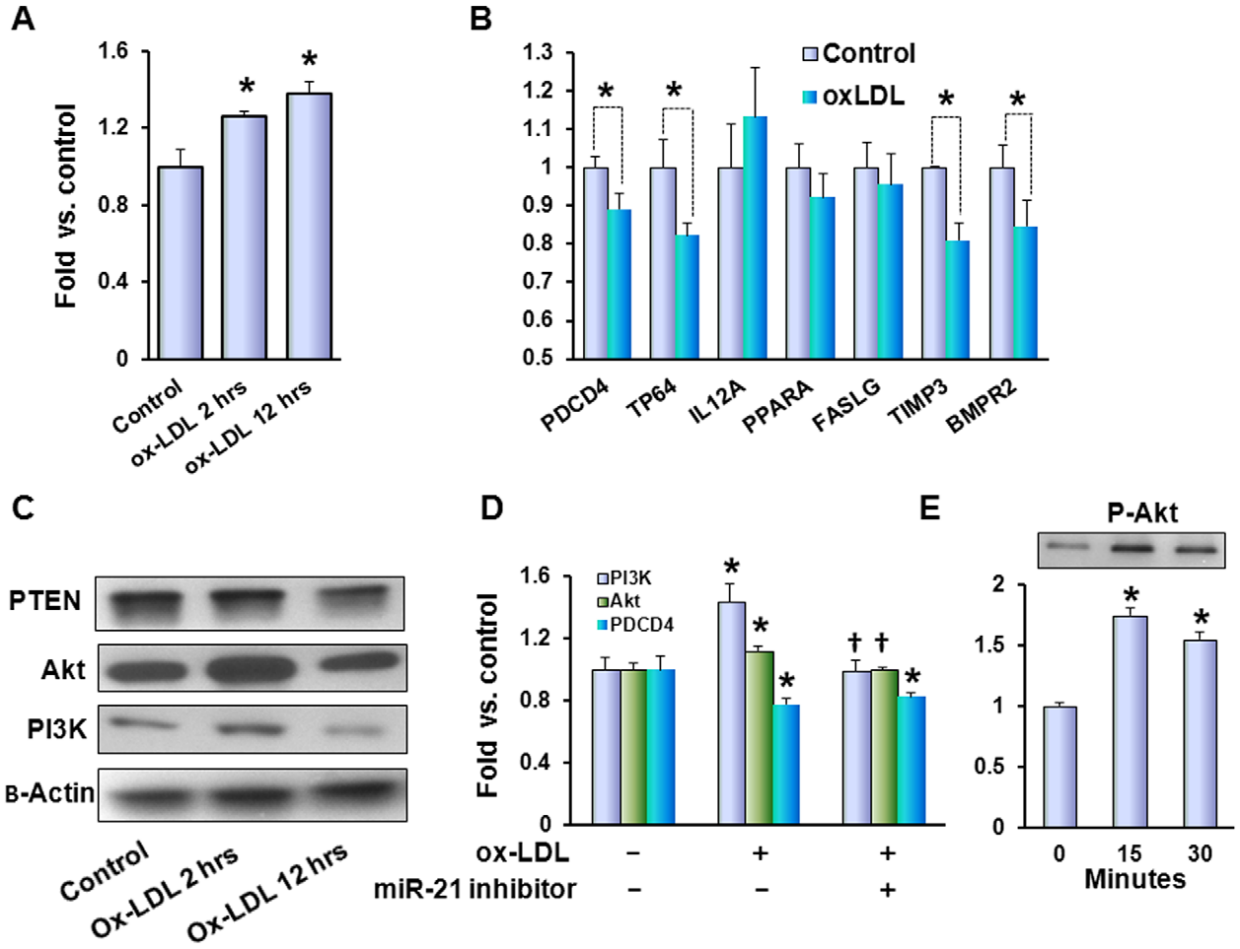
Ox-LDL increases expression of miR-21 accompanied with PI3K/Akt upregulation and inhibition of target genes. (**A**) – qPCR analysis for expression of hsa-miR-21; (**B**) – qPCR analysis for expression of hsa-miR-21 target genes in control (white columns) and ox-LDL treated (20 µg/ml, 12 hours, black columns) cells; (**C**) – Western blot for PTEN, Akt and PI3K; (**D**) - qPCR analysis for expression of PI3K, Akt and PDCD4 genes in control cells and miR-21 inhibitor transfected cells treated with ox-LDL; (**E**) – Western blots for phospho-Akt within short (30 min) exposure to 20 µg/ml ox-LDL with corresponding graph depicting relative densities of bands normalized for β-actin in relation to control (fold change); (*) – significant difference compared to control. (†) - Significant difference (p<0.05) compared to ox-LDL treated non-transfected cells.

Expression of miR-21 increased upon treatment of cells with 20 µg/ml ox-LDL and was accompanied by downregulation of several miR-21 target genes (Figure 4A and B). The stimulation of miR-21 became evident at 2 hours and coincided with an increase in Akt and PI3K content, while decline of PTEN content became noticeable only after 12 hours of ox-LDL treatment (Figure 4C). Notably, transfection of MCF10A cells with miR-21 inhibitor cells cancelled the effects of ox-LDL on PI3K and Akt on transcriptional level (Figure 4D). Activation of PI3K/Akt pathway was further confirmed by quick, within 15 minutes phosphorylation of Akt (Figure 4E).

## Discussion

We have shown that human mammary epithelial cells (MCF10A) express scavenger receptors and internalize ox-LDL, and that exposure to ox-LDL stimulates CD36 and, albeit to a lesser extent, LOX-1 expression. MCF10A cells appear to have greater tolerance to ox-LDL than endothelial cells and exhibit signs of distress at 50 µg/ml, whereas stimulation of tube formation by endothelial cells is compromised upon exposure to as little as 10 µg/ml ox-LDL [17].

One of the hallmarks of perturbed lipid metabolism is increase in circulating ox-LDL concentrations [18], [19] resulting from increased ROS production from various sources including myeloperoxidase, NADPH oxidase, xanthine oxidase, cytochrome 450, nitric oxide synthase and mitochondrial complexes I and III as well as lipoxygenases and phospholipases. In our studies, ox-LDL provoked relatively rapid (within 2 hours of exposure) stimulation of subunits of NADPH oxidase P22^phox^ and P47^phox^. The increase in NADPH oxidase was followed by (within 12 hours of exposure), upregulation of lipoxygenases-12 and -15. Activation of NADPH oxidase and resulting increase in superoxide production is a part of a typical reaction of vascular cells to ox-LDL [20], [21]. The concomitant increase of SOD1 content In MCF10A is suggestive of a compensatory response to elevated oxidative stress. It should also be noted that several constituents of ox-LDL such as MDA and 4-HNE have been shown to have direct mutagenic and carcinogenic properties [22], [23].

In vascular cells, lipoxygenases have been shown to be primary enzymatic modifiers of LDL responsible for about 70–80% of LDL oxidation [24]–[26]. Upregulation of lipoxygenase-12 and -15 combined with stimulation of scavenger receptors observed in this study create the possibility for local overproduction and consumption of ox-LDL and, thus, amplification of ox-LDL signaling.

Available information on contribution of lipoxygenases to oncogenic transformation and tumorigenesis is controversial and suggests mutually exclusive effects for many of their metabolites. Lipoxygenase-12 has been shown to be overexpressed in variety of cancers [27], [28]. In breast cancer, expression of lipoxygenase-12 cells was significantly higher compared to adjacent tissues [29] and transfection of poorly invasive MCF7 cells with the enzyme resulted in their rapid growth in nude mice accompanied with enhanced proliferation and reduced apoptosis [30]. In another study, expression of lipoxygenase-12 positively correlated with susceptibility to transformation of mouse epidermal cells JB6 [31]. The product of lipoxygenase-12, 12-hydroxy eicosatetraenoic acid (12-HETE) displayed mitogenic properties in breast cancer cells and its blockade by inhibitors resulted in downregulation of Bcl2, release of cytochrome c and activation of caspases [32].

In contrast, lipoxygenase-15-1 and lipoxygenase-15-2 are typically downregulated in cancer cells in vivo and in vitro [33], [34]. The effects of lipoxygenases on epithelial and cancer cells in published reports vary; for example, overexpression of lipoxygenase-15-1 in colon carcinoma cells Caco-2 activated MAPK pathway, decreased p21 expression and stimulated proliferation [35], whereas similarly processed colorectal cells HCT-116 produced smaller tumors in a nude mice [36]. The product of lipoxygenase-15-1, a metabolite of linoleic acid 13-S-hydroxyoctadecadienoic acid (13-S-HODE) inhibited proliferation and induced cell arrest in transformed colonic cell line [37]. Similarly, lipoxygenase-15-2 expression or utilization of its metabolite 15-S-Hydroxyeicosatetraenoic acid (15(S)-HETE) had negative effect on progression of prostate epithelial cells [38].

We observed activation of PI3K/Akt, MAPK and NF-kB pathways in MCF10A cells in response to ox-LDL treatment which is consistent with findings reported for vascular endothelial cells [39]-[41]. Stimulation of the MAPK signaling pathway due to overexpression of its activators RTKs, Ras, and Raf is a common occurrence in tumors [42]-[44]. In breast cancer cells, activation of p44/42 MAPK has also been found to be directly responsible for loss of estrogen receptors and, hence, more aggressive phenotype [45].

In our recent microarray study on ox-LDL transcriptome in human endothelial cells (manuscript in preparation) we have determined that ox-LDL stimulates hsa-miR-21- a miRNA with well-established involvement in the pathobiology of both cardiovascular diseases and cancer [46]. Mir-21 is upregulated in many tumors [47], including breast cancer [48], [49], and its role as a genuine oncogene has been recently shown in mice when inducible overexpression of miR-21 led to dramatic increase in hematological malignancies followed by their complete reversal after returning animals to a normal miR-21 expression mode [50]. The actions of miR21 are attributed to the central role of its primary target PTEN (phosphatase and tensin homolog deleted from chromosome ten) in Akt signaling. PTEN antagonizes PI3K by cleaving its major product, lipid PtdIns (3,4,5)*P_3_*, and thus preventing activation of downstream Akt signaling cascade. In the present study, ox-LDL caused mild stimulation of miR-21 accompanied with decline in PTEN and reciprocal increases in concentrations of PI3K and Akt. These results coupled with downregulation of several miR-21 target genes as well as lack of PI3K and Akt transcriptional activation in response to ox-LDL in cells transfected with miR-21 inhibitor indicate that miR-21, indeed, participates in shaping ox-LDL action by triggering PI3K/Akt cascade.

In summary, our data suggest that mechanisms associated with LDL oxidation and internalization of ox-LDL by mammary epithelial cells may play a significant role in dyslipidemia-mediated enhanced susceptibility to breast cancer via variety of mechanisms including activation of NADPH oxidase and activation of pro-proliferative and pro-inflammatory signaling. Also, we show the first time that ox-LDL stimulate miR-21 - one of the true oncogenes intimately involved in carcinogenesis.
